# Mechanistic Insights of Polyphenolic Compounds from Rosemary Bound to Their Protein Targets Obtained by Molecular Dynamics Simulations and Free-Energy Calculations

**DOI:** 10.3390/foods12020408

**Published:** 2023-01-14

**Authors:** Samo Lešnik, Marko Jukič, Urban Bren

**Affiliations:** 1 Laboratory of Physical Chemistry and Chemical Thermodynamics, Faculty of Chemistry and Chemical Engineering, University of Maribor, Smetanova ulica 17, SI-2000 Maribor, Slovenia; 2 IOS, Institute of Environmental Protection and Sensors, Beloruska ulica 7, SI-2000 Maribor, Slovenia; 3 Faculty of Mathematics, Natural Sciences and Information Technologies, University of Primorska, Glagoljaška 8, SI-6000 Koper, Slovenia

**Keywords:** rosemary, carnosic acid, carnosol, rosmanol, rosmarinic acid, polyphenols, molecular docking, molecular dynamics simulations, linear interaction energy calculations, water-mediated hydrogen-bonds, HIV-1 protease, K-RAS protein, factor X

## Abstract

Rosemary represents an important medicinal plant that has been attributed with various health-promoting properties, especially antioxidative, anti-inflammatory, and anticarcinogenic activities. Carnosic acid, carnosol, and rosmanol, as well as the phenolic acid ester rosmarinic acid, are the main compounds responsible for these actions. In our earlier research, we carried out an inverse molecular docking at the proteome scale to determine possible protein targets of the mentioned compounds. Here, we subjected the previously identified ligand–protein complexes with HIV-1 protease, K-RAS, and factor X to molecular dynamics simulations coupled with free-energy calculations. We observed that carnosic acid and rosmanol act as viable binders of the HIV-1 protease. In addition, carnosol represents a potential binder of the oncogene protein K-RAS. On the other hand, rosmarinic acid was characterized as a weak binder of factor X. We also emphasized the importance of water-mediated hydrogen-bond networks in stabilizing the binding conformation of the studied polyphenols, as well as in mechanistically explaining their promiscuous nature.

## 1. Introduction

**Figure 1:**
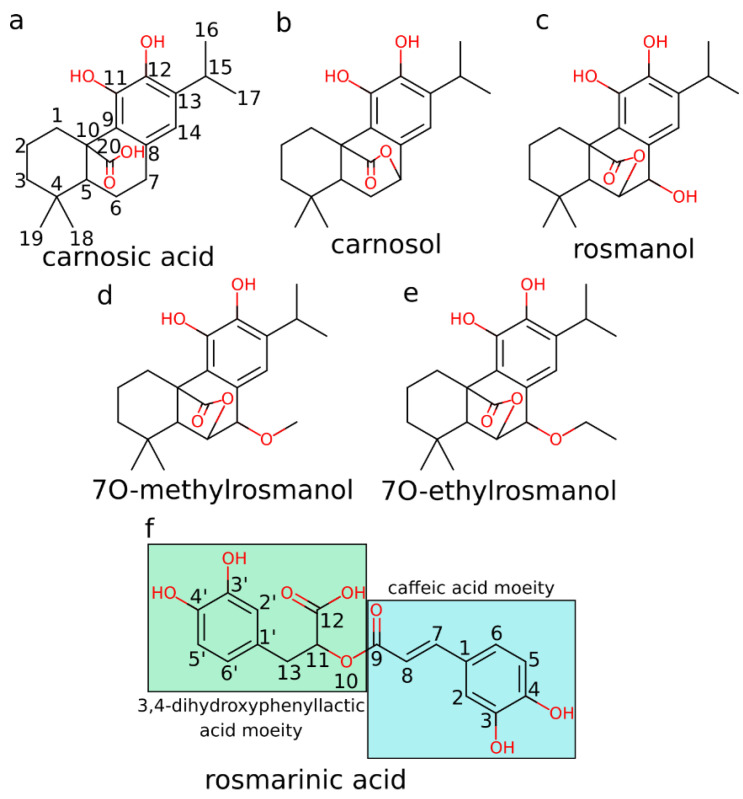
The 2D structures and atom numbering of investigated compounds: (**a**) carnosic acid, (**b**) carnosol, (**c**) rosmanol, (**d**) 7O-methylrosmanol, (**e**) 7O-ethylrosmanol, and (**f**) rosmarinic acid.

Natural products play a crucial role in the development of new drugs, as 30% of the drugs developed in the last decades resulted from unmodified natural products or their derivatives [1]. An important subgroup of natural products represents polyphenols, secondary plant metabolites, which form a large group of diverse compounds containing one or more aromatic rings with one or more hydroxyl groups attached to them [2]. Rosemary (*Rosmarinus officinalis* L.) represents an evergreen shrub that exhibits numerous beneficial properties for human health [3]. In particular, studies indicate that the bioactive polyphenols present in rosemary yield antioxidative [4], anti-inflammatory [5], antimicrobial [6], antidiabetic [7], and cognitive-enhancing [8,9] effects. In addition, rosemary extracts display promising anticarcinogenic activities in vitro [10,11,12] and in vivo [13,14]. Polyphenols carnosic acid, carnosol, rosmanol, and rosmarinic acid are most frequently mentioned in connection with the beneficial pharmacological activities of rosemary [3] (Figure 1a–c,f). Carnosol, carnosic acid, and rosmanol represent diterpenes with similar structures. They consist of the main abietane scaffold, a fused six-membered tricyclic ring system, one of these rings being aromatic. Carnosic acid is the main constituent of rosemary and can be present in amounts of up to 4% weight of the dried leaves [15]. Rosmarinic acid is a polyphenolic ester of caffeic acid and 3,4-dihydroxyphenyllactic acid [16]. Dried rosemary leaves represent a good source of rosmarinic acid, containing around 10 mg/g of the substance [17]. 

In this study, we employed a suite of in silico approaches combining molecular docking, molecular dynamics (MD) simulations, and linear interaction energy (LIE) free energy calculations to further evaluate the binding of rosemary compounds to protein receptor structures identified in our previous inverse molecular docking study [18].

In the inverse docking study mentioned above, we identified numerous human, bacterial, viral, and parasitic targets to which rosemary compounds can potentially bind [18]. The anticarcinogenic mechanism of the diterpenes could be explained by the fact that they bind to proteins that play a role in cancer cell growth, including the Kirsten rat sarcoma viral oncogene homolog (K-RAS), pyruvate kinase M2, peroxisome proliferator-activated receptor delta, tubulin, phospholipase A2, and vascular endothelial growth factor receptor 2. The anticarcinogenic effect of rosmarinic acid can be explained by its binding to matrix metalloproteinase-3, glutamate dehydrogenase 1, glutaminase, and phospholipase A2. Phospholipase A2 is also involved in inflammation-related diseases; thus, its inhibition could also be beneficial in arthritis, coronary heart disease, or dementia. The binding of rosemary diterpenes to glycogen phosphorylase, which facilitates glucose production, may confer a beneficial effect in the treatment of type II diabetes. In addition, rosemary diterpenes may have antiviral activities by binding to the proteases of HIV-1 and HIV-2, as well as to the hemagglutinin HA1 of the influenza virus.

Our inverse docking study suggests that the antibacterial activity of the diterpenes can be explained by their binding to the enzyme glucosamine fructose-6-phosphate aminotransferase in *Escherichia coli*, which catalyzes a critical step in hexosamine metabolism responsible for bacterial growth. Moreover, they can bind to the Eis protein of *Mycobacterium tuberculosis*, which is responsible for its resistance to aminoglycosides.

The diterpenes studied also showed binding to the aspartate carbamoyltransferase of two pathogenic parasites—*Plasmodium falciparum* and *Trypanosoma cruzi*, which cause malaria and Chagas disease, respectively. Inhibition of this enzyme results in the parasites’ inability to produce pyrimidines, limiting their production of nucleic acids.

Rosmarinic acid could bind to factor X, reducing excessive blood clotting and preventing diseases such as heart attacks and ischemic strokes. Moreover, rosmarinic acid could also act against parasites, as its binding to the farnesyl pyrophosphate synthase of *Leishmania major* could prevent the synthesis of ergosterol and thus be potentially applicable in the treatment of zoonotic cutaneous leishmaniasis.

In this paper, we focus on the protein targets identified in the aforementioned inverse molecular docking study [18] that are important for human health, namely, human immunodeficiency virus 1 (HIV-1) protease, K-RAS, and coagulation factor X. We selected HIV-1 protease, because of its need for novel inhibitors due to increasing resistance to current drugs [19]. K-RAS represents a crucial oncogene, implicated in a wide variety of cancers [20,21], and rosemary extracts were found to reduce its expression, whereas factor X stands at the forefront of the coagulation cascade which is connected to cardiovascular diseases—the leading cause of death in the Western world [22].

The HIV-1 protease is a retroviral aspartyl protease that is crucial to the life cycle and replication of the HIV virus because it cleaves the gag and gag-pol polyproteins into mature functional proteins [23]. As a component of a highly effective antiretroviral therapy, small-molecule inhibitors of the HIV-1 protease are essential in the treatment of acquired immunodeficiency syndrome (AIDS). However, due to the mentioned increasing resistance of HIV-1 to antiretroviral drugs, new inhibitors of HIV proteases are urgently needed [19]. HIV-1 protease represents a symmetrical homodimeric protein with its binding site located within the cavity formed by the two dimers. Each chain possesses a catalytic aspartate residue (Asp25) and a "flap" folding over the bound substrate. An experimental study performed by Pariš et al. [24] demonstrated that the phenolic diterpene from rosemary rosmanol, and its semisynthetic derivatives 7-O-methylrosmanol and 7-O-ethylrosmanol strongly inhibit HIV-1 protease, whereas carnosol exhibited less pronounced inhibitory effects towards the enzyme. Recently, multiple short MD simulations were used to investigate the drug resistance mechanism of V32I, I50V, and I84V mutations toward amprenavir [25]. Binding free energies calculated using the MM-GBSA method suggested that the decrease in binding enthalpy with the concurrent increase in binding entropy induced by the mutations V32I, I50V, and I84V confers drug resistance.

K-RAS is an example of a GTPase that relays signals from the outside of the cell to the nucleus. It is a component of the rat sarcoma/mitogen-activated protein kinase (RAS/MAPK) pathway, and it transmits signals that promote cell proliferation, growth, and differentiation. Due to the fact that it is the most often mutated oncogene in lung, colon, and pancreatic cancers, it is of the utmost clinical significance [20]. Although various attempts have been made to create K-RAS inhibitors, few have been successful [26]. The difficulties in drug development are primarily brought on by the absence of a clearly defined binding pocket and the high affinity of the K-RAS binding site for guanosine triphosphate (GTP), with which potential drug ligands struggle to compete or, when they can, typically display a poor selectivity. Nevertheless, there has recently been some advancement in the use of small-molecule ligands to modulate K-RAS. A potent inhibitor of the oncogenic K-RAS G12C mutant was created by Fell et al. [27], which causes the formation of a new binding pocket close to the nucleotide (GTP) binding site. By arresting the enzyme in its inactive state, the binding to this new pocket inhibits the signal transduction. Moreover, our previous inverse molecular docking study demonstrated that rosemary diterpenes could bind to this induced binding site [18]. Although there is no direct experimental evidence of rosemary compounds binding to the main or induced binding site of K-RAS, it was shown that rosemary extracts downregulate K-RAS gene expression levels in colon cancer tissue [21]. Previously, Issahaku et al. [28] applied the single-trajectory protocol MM/PBSA method to evaluate the associating energies accompanying the binding of the K-RAS switch II inhibitor AMG510 to different mutations that occur in the switch II binding pocket. They demonstrated that any mutations differing from G12C strongly reduce the binding affinity of AMG510 to K-RAS.

In the coagulation cascade, factor X functions as an essential enzyme that, when activated by hydrolysis, transforms prothrombin into the active form, which then transforms soluble fibrinogen into insoluble fibrin strands [29]. The role of factor X is particularly important, because it represents the first enzyme, where the intrinsic and extrinsic coagulation pathways converge. Currently, drug manipulation of the coagulation cascade is crucial in the clinical practice, as reducing excessive coagulation is critical for the prevention of cardiovascular diseases such as myocardial infarction and ischemic stroke, which are among the leading causes of death and disability in the Western world [22,30]. Oral inhibitors of factor X, such as rivaroxaban, are already successfully used in clinical practice [31]. We are not aware of any existing experimental studies demonstrating the binding of rosemary compounds to factor X. However, previously, Genheden and Ryde [32] used a series of MM/PB(GB)SA approaches to estimate the binding affinity of nine 3-amidinobenzyl-1H-indole-2-carboxamide inhibitors.

## 2. Materials and Methods

### 2.1. Target Preparation and Docking

Molecular docking was performed using the recently developed CmDock software (v. 0.1.4; https://gitlab.com/Jukic/cmdock accessed on 20 September 2022) [33], which represents a fork of RxDock/rDock with modern tool additions, optimizations, and adaption for modern hardware and software [34]. We applied the experimental co-crystallized ligands to define the docking grid 5 Å around the reference ligand heavy atoms. All structures were downloaded from the RCSB Protein Data Bank (PDB). For HIV-1 protease, the structure with PDB ID 5YOK, for K-RAS, the structure with PDB ID 4LUC, and, for factor X, the structure with PDB ID 2JKH were employed. Explicit water molecules were not considered during molecular docking. With 100 runs utilizing DOCK.prm settings, we used a sample technique that included three stages of genetic algorithm search, low-temperature Monte Carlo, and simplex minimization phases, as well as scoring using the rDOCK (SF3) scoring function [34]. The sampling and scoring were validated against protein [34,35] and RNA [34,36] targets, and their performance was superior to similar open-source software [37].

### 2.2. Molecular Dynamics Simulations and LIE Calculations

The protein–ligand complexes were prepared for MD simulations using the Chemistry at Harvard Macromolecular Mechanics graphical user interface (CHARMM-GUI) [38] by implementing the structures obtained from the molecular docking. Prior to the MD simulations, the complexes were solvated in cubes of TIP3P water molecules (15 Å padding using periodic boundary conditions) with 0.15 M NaCl. Each time, the appropriate number of Na^+^ or Cl^−^ ions was added to render the overall system neutral. The protonation states of ionizable amino-acid residues were set as standard in Chemistry at Harvard Macromolecular Mechanics (CHARMM), i.e., Asp/Glu are negatively charged, Arg/Lys are positively charged, and His residues are singly protonated at the N1δ atom. The standard CHARMM36 forcefield parameters for proteins were used [39,40], supplemented by the CHARMM36-WYF set, which improves the description of potential cation–π interactions [41]. Forcefield parameters for all four ligands (carnosic acid, carnosol, rosmanol, and rosmarinic acid) were determined using the automated ParamChem web server [42]. We are fully aware of the drawbacks of using automated methods to determine forcefield parameters for drug-like small molecules; however, due to the low overall reported nonbonded penalties for the majority of sterically accessible ligand atoms, as well as due to the reported low penalties for bonded interactions corresponding to flexible moieties, we consider these parameters reliable for our purposes. Moreover, we emphasize that, as the LIE represents an end-state method, its results are much less affected by any forcefield corrections. The systematic errors addressed by these corrections affect both end-states and, therefore, cancel out, when their relative differences are calculated [43,44,45].

The coordinate files of proteins and water molecules were combined, and 50 steps of steepest descent and 50 steps of adopted basis Newton–Raphson energy minimizations were carried out to remove any potential steric clashes that may occur and to optimize the atomic coordinates of the complexes. The protein was then equilibrated at 310.15 K using the HOOVER thermostat and an integration timestep of 1 fs during a brief MD simulation. The NVT ensemble’s equilibration molecular dynamics took 0.125 ns to complete. This was followed by five independent production runs of 30 ns. Production runs were carried out in the NPT ensemble with periodic boundary conditions applied, with the timestep of 2 fs and the HOOVER thermostat and barostat set to 310.15 K and 1 bar, respectively. Van der Waals interactions were cut off between 10 and 12 Å using the force switch method (VFSWIt). The electrostatic potential used the force shifting method (FSHIft) again with a cutoff of 12 Å. The particle mesh Ewald summation [46] was applied to calculate long-range electrostatic interactions. Bonds involving hydrogen atoms were constrained using the SHAKE algorithm. The ligand–water MD simulations were initiated from the MP2/6-31G optimized ligand conformations. 

Using MD simulations, binding affinities of ligand–receptor complexes were calculated with the LIE method. LIE represents a way to combine molecular mechanics calculations with experimental data to build a model scoring function for explicitly assessing ligand–protein binding free energies [47]. The ligand binding free energy is calculated using Equation (1).

(1) $$\Delta G_{\mathrm{bind}}=\alpha \overset{N}{\underset{i}{\sum }}\Delta V_{i}^{\mathrm{vdW}}+\beta \overset{N}{\underset{i}{\sum }}\Delta V_{i}^{\mathrm{coulomb}},$$

where ΔG is the difference in potential energy between the ligand’s bound and unbound (in water alone) states. α and β form the empirically obtained LIE parameters determined by comparing the calculated and experimentally measured binding affinities. Their values were optimized by Åqvist et al. [47]. Particularly important for polyphenolic structures is the subsequently developed differentiation by Hansson et al. [48], which classifies ligands into four groups: (a) charged (β = 0.5), (b) dipolar without hydroxyl groups (β = 0.43), (c) dipolar with one hydroxyl group (β = 0.37), and (d) dipolar with two or more hydroxyl groups (β = 0.33). However, this classification was developed using very simple compounds (e.g., ethylene glycol for group (d)), and subsequent studies have shown that parameter values are specifically dependent on the ligand and protein target, as well as the forcefield parameters used and can vary widely across different studies [49,50,51,52,53,54]. Some, therefore, consider α and β in a QSAR-like manner, i.e., they treat them as completely free parameters, where their values can even be negative [53,55]. However, in the absence of suitable experimental binding studies for K-RAS and factor X systems, we applied the parameters optimized by Hansson et al. [48]. To parameterize the α- and β-values for the HIV-1 protease system, we used the IC_90_ values reported by Pariš et al. [24]. To transform the IC_90_ values to ΔG_bind_, we applied the following expression:

(2) $${\Delta G}_{\mathrm{bind}}=\mathrm{RT}\cdot \ln\left({\mathrm{IC}}_{90}\right).$$

Only three IC_90_ values, for rosmanol, 7O-methylrosmanol, and 7O-ethylrosmanol were provided in the aforementioned study; for carnosol and dimethylcarnosol, the authors only reported an IC_90_ value >10 μg/mL, whereas carnosic acid is believed to be spontaneously metabolized before the binding experiment, such that its measured IC_90_ value cannot be used directly. We are aware of the very limited dataset on the basis of which we obtained the optimized α- and β-values. However, until further experimental measurements of rosemary diterpenes binding to HIV-1 protease are performed, a better parameterization is simply not possible. By fitting the average LIE interaction energies to the experimental ΔG_bind_ values using an in-house Python script implementing the *scipy.optimize* package [56], we obtained the following parameter values: α = 0.74 and β = 0.26, which are consistent with the fact that HIV-1 protease binding occurs mainly through hydrophobic interactions [57]. Previously, Huang et al. [52], using the CHARMM22 forcefield [58], reported for HIV-1 protease an α value of 0.17 and a much lower β value of 0.02. In this work, we also present the parameters from Huang et al. [52] for comparison purposes, as well as the standard empirical parameters developed by Hansson et al. [48].

Following the initial molecular dynamics equilibrations, five independent 30 ns molecular dynamics production runs of nine different protein–ligand complexes and six 200 ns MD production runs of ligands in water only (Figures S1 and S2) were performed to obtain the average van der Waals and electrostatic interaction energies between the ligand and its environment (totaling to over 2.5 μs of simulation time). The recent scientific literature shows that producing several independent shorter molecular dynamics trajectories clearly outperforms longer molecular dynamics trajectories in terms of conformational space sampling [59,60,61]. From these simulations, we calculated the average electrostatic and vdW interaction energies between 5 and 30 ns, since during this time interval the RMSD and energy values converged. No major conformational changes or chain movements could also be observed during this time interval. The potential energy values were determined using the *interaction* function in CHARMM. Compared to MM/PBSA, the LIE method is considered suitable for the calculation of a larger number of ligand binding free energy values, as it produces relevant results even with short simulation times [48,62,63]. 

Root-mean-square deviations were calculated with the *MDAnalysis* Python library [64], and H-bonding networks were analyzed implementing the recently developed *Bridge2* software [65,66]. The interactions between protein and ligands were identified using the automated Protein–Ligand Interaction Profiler (PLIP) [67,68]. Each system was subsequently visually checked in order to confirm the accuracy of the predictions. 

In addition, we used the *MDAnalysis* Python library to obtain interaction occupancies calculated on the basis of all atoms at the interface between protein and ligand within 5.5 Å of each other, resulting in a possibility of occupancies being greater than 100%. Bridge2, on the other hand, counts water-mediated H-bonds only, where H-bonds between a ligand and a single amino-acid residue can only be counted once. Consequently, the highest value obtained can be 100%, allowing for a more detailed and deeper analysis of the interaction profile.

## 3. Results and Discussion

### 3.1. HIV-1 Protease Molecular Docking

**Figure 2:**
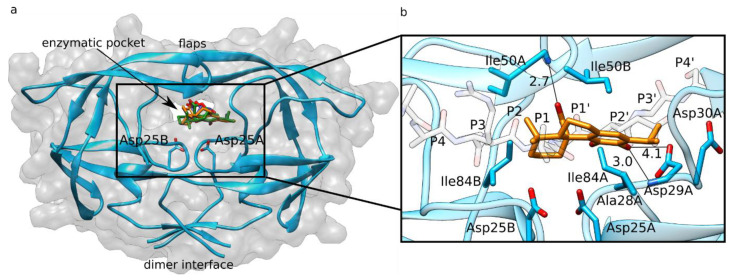
Overview of docked complexes. (**a**) Overlap between carnosic acid (orange), carnosol (green), and rosmanol (purple) in the binding pocket of HIV-1 protease (blue ribbons and gray surface). The enzymatic pocket, flaps, dimer interface, and catalytic aspartate are shown. (**b**) The docked pose of carnosic acid. Carnosic acid, as well as carnosol and rosmanol, form numerous hydrophobic interactions with the annotated amino-acid residues, as well as hydrogen bonds (black lines with distances in Å) with the main chain atoms and the side-chain of Asp29A. Carnosic acid also forms a hydrogen bond between its acidic group and the main chain of Ile50A. For orientation, we also depict residues Asp25A and Asp25B, which do not form direct interactions with diterpenes. In transparent sticks with gray carbons, we display the native peptide substrate (based on the PDB ID: 1F7A structure), according to which the subpockets are defined. This figure is based on the HIV-1 protease crystal structure with the PDB ID 5YOK.

The molecular docking poses determined by CmDock with the best results for carnosic acid, carnosol, and rosmanol (Supporting Materials Table S1) were used as starting structures for the subsequent MD simulations. Within the catalytic pocket formed between the two monomers of HIV-1 protease, the docked poses of all three diterpenes exhibited a high overlap (Figure 2a).

The HIV-1 protease binding pocket can be divided into eight subunits as a function of the amino-acid residues occupying different parts of the enzymatic pocket. According to our molecular docking study, all diterpenes bind to the catalytic pocket near the Asp25 residues (Figure 2a) and specifically occupy the P1, P1′, and P2′ subpockets (Figure 2b) [69]. The three diterpenes exhibit numerous hydrophobic interactions with Ala28A, Asp30A, Ile50A, Ile84A, Ile50B, and Ile84B (Figure 2b). The phenol group of the three diterpenes forms hydrogen bonds with the main and side-chains of Asp29A. The carboxylic acid moiety of carnosic acid forms a hydrogen bond with the main chain of Ile50A, while carnosol and rosmanol form an analogous hydrogen bond with their carbonyl groups. The diterpenic ligands of course form less interactions compared to the native crystal ligand (PDB ID 8Z0), which is a peptidomimetic, occupying a larger volume within the binding site, also reaching the P1–P4 subpockets (Figure S3). Specifically, the crystal ligand forms hydrophobic interactions with Leu23A, Ala28A, Ile47B, Ile47A, Gly49A, Ile50A, and Ile84B. Moreover, it forms hydrogen bonds with Gly27A, Asp29A, Asp30A, Gly48A, and Gly49A.

The additional starting structures of 7O-methylrosmanol (Figure 1d) and 7O-ethylrosmanol (Figure 1e) required for the parametrization of the LIE calculations were obtained by adding 7O-methyl and 7O-ethyl moieties to the best docked rosmanol pose, respectively.

### 3.2. HIV-1 Protease MD Simulations and LIE Calculations

**Figure 3:**
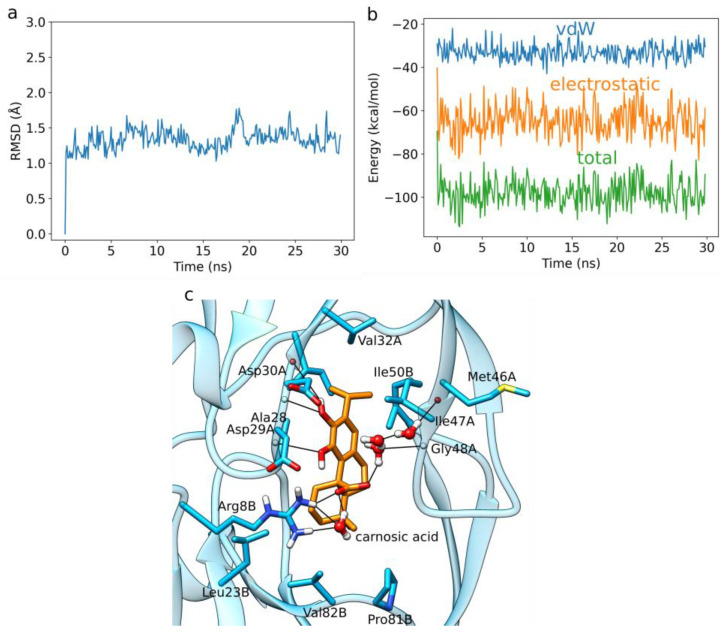
MD simulation of carnosic acid bound to HIV-1 protease. (**a**) RMSD of the protein backbone of the analyzed MD simulation. (**b**) Interaction energy analysis describing the binding between carnosic acid and HIV-1 protease as observed in the MD simulation. (**c**) Carnosic acid forms a stable H-bond network in the HIV-1 protease binding site. Hydrogen bonds are shown with black lines. The remaining amino-acid residues displayed with sticks form hydrophobic contacts. We show stable H-bonds and hydrophobic contacts with occupancy of at least 50%.

We performed five independent 30 ns long MD simulations of HIV-1 protease in complex with each diterpene ligand. Below, we present the binding pattern observed for carnosic acid, which exhibited the best LIE binding free energies using our newly obtained α- and β-parameters. The RMSD plot of the protein backbone corresponding to a prototypical MD run, as well as the averaged protein and ligand RMSD plots and binding energy timeseries of all five replicas point to a converged dynamics (Figure 3a, Figures S4a–e, and S5a–e). The plot of the total interaction energy displays, for carnosic acid, a fluctuation around a stable average value of about −100 kcal/mol after the initial 5 ns of the MD simulation (Figure 3b).

Carnosic acid exhibits a change in the binding position, mainly due to the formation of numerous stable water-mediated H-bond bridges observed during the MD simulations. In particular, carnosic acid forms with its acidic moiety both direct and water-mediated H-bond interactions with the side-chain of Arg8B (Figure 3c). It also forms an H-bond mediated by a single water molecule with the backbone of Gly48A and by three water molecules with the backbone of Met46A. One catechol OH group of carnosic acid forms a direct H-bond with the side-chain, as well as the backbone of Asp29A, while the other forms a direct H-bond to Asp30A. Several hydrophobic interactions were detected within the MD simulations, with Gly27A, Ala28A, Val32A, Ile47A, Leu23B, Ile50B, Pro81B, and Val82B. An exhaustive interaction profile averaged across all five replicas for each ligand is presented in Table S5. 

For the LIE calculations, parameters obtained by fitting our free energy calculations to the experimental IC_90_ values using rosmanol, 7O-methylrosmanol, and 7O-ethylrosmanol were used. We additionally present results obtained with α- and β-parameters based on the work of Huang et al. [52], and (b) based on Hansson et al. [48] (Table S2).

The results display a similar binding affinity for carnosic acid, 7O-methylrosmanol, and rosmanol, albeit the latter with a relatively high standard deviation, again especially due to Coulomb interactions (Table 1). Meanwhile, carnosol displays slightly poorer binding, with a high observed standard deviation of the Coulomb contribution.

Carnosic acid, carnosol, and rosmanol undergo extensive metabolic reactions in vivo. They are, for example, able to interconvert via the carnosic acid quinone intermediate [70]. The diterpenes may also be metabolized in vivo and in vitro by oxidation, glucuronidation, and methylation reactions, and in vivo especially in intestinal and liver tissues [71,72]. In animal models using rats and mice, it was found that, after their oral administration, the major species appear to be glucuronidated diterpenic metabolites, while the aglyconic forms continue to be detected in plasma [72,73,74,75]. 

To obtain a complete interaction profile of the investigated rosemary natural products, the addition of their major metabolites described in the scientific literature would be beneficial. However, we decided to perform our study only using aglycone structures, since experimental inhibitory values for HIV-1 protease have been reported only for them [24]. We are aware of the limitations pertinent to studying only the aglycone structures, since they do not necessarily represent the predominant forms in in vivo systems following oral administration. However, due to the extremely complex metabolic profiles of carnosic acid, carnosol, and rosmanol, we focused on the aglycones, whereas we plan to extend our in silico methods to their major metabolites in future studies.

### 3.3. K-RAS Docking

**Figure 4:**
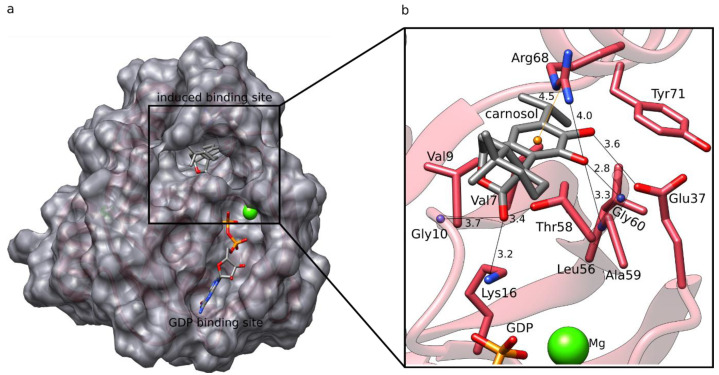
Overview of the docked complex with K-RAS. (**a**) K-RAS (pink ribbons and gray surface) with the depicted GDP binding site with GDP bound and the induced binding pocket with carnosol docked. Both ligands exhibit gray carbons. Magnesium ions are shown as green spheres. (**b**) The docked pose of carnosol in the induced binding site forms numerous hydrophobic interactions, as well as H-bonds (black line with distance in Å). Moreover, it makes a cation–π stacking interaction with Arg68 (orange line). The figure is based on the crystal structure with the PDB ID 4LUC.

In our previous inverse docking study [18], we demonstrated that rosemary diterpenes can successfully bind to the induced binding pocket previously reported by Fell et al. [27] (Figure 4a). Despite the fact that no diterpene has been found to directly bind to K-RAS, rosemary extracts have been proven to reduce K-RAS expression in colorectal cancer cells [21]. This raises the intriguing possibility that rosemary diterpenes could attack the protein in two ways: by lowering its expression and by directly inhibiting it.

Among the three diterpenes, carnosol exhibited the best docking score (Table S3). Analyzing its binding pattern (Figure 4), we observe that it can form several types of interactions within the induced binding site of K-RAS. It forms hydrophobic interactions with Val7, Val9, Leu56, Thr58, Arg68, and Tyr71. Hydrogen bonding is formed between carnosol’s catechol groups and the main chains of Ala59 and Gly60, as well as the side-chains of Glu37 and Arg68. The ester group of carnosol forms additional H-bonding interactions with the main chain of Gly10, as well as the side-chains of Lys16 and Thr58. Lastly, a cation–π interaction is formed between the catechol ring of carnosol and Arg68. The number of interactions is similar to the native crystal covalent inhibitor (PDB ID 4LUC, PDB ligand ID 20G) (Figure S6), as the latter forms hydrophobic interactions with Val9, Thr58, and Arg68, hydrogen bonds with the main chain of Gly60 and Gln99, and a halogen-bonding interaction with the main chain of Tyr96.

### 3.4. K-RAS MD Simulations and LIE Calculations

**Figure 5:**
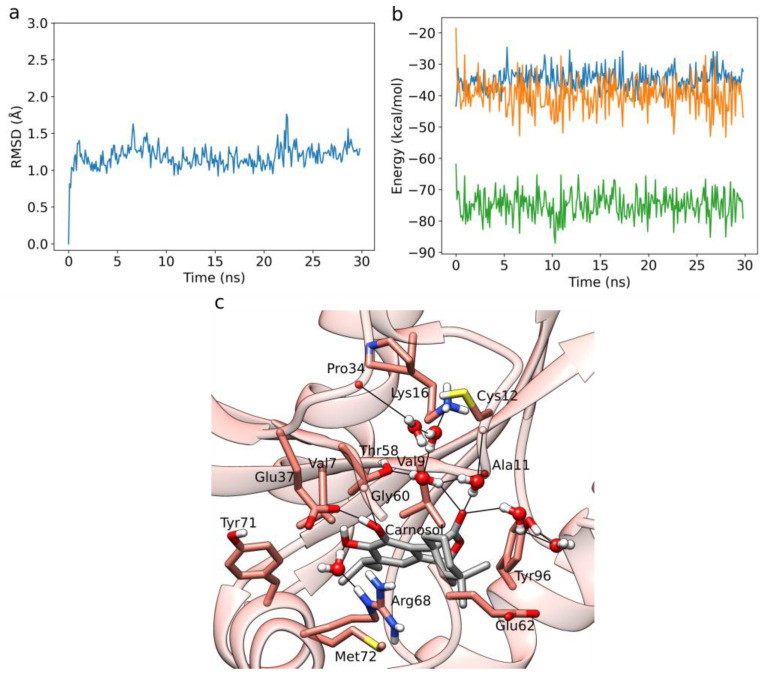
MD simulation of carnosol bound to K-RAS. (**a**) RMSD of the protein backbone of the analyzed MD simulation. (**b**) Interaction energy analysis describing the binding between carnosol and K-RAS as observed in the MD simulation. (**c**) Carnosol forms a stable H-bond network in the K-RAS binding site. Hydrogen bonds are shown with black lines. The remaining amino-acid residues displayed with sticks form hydrophobic contacts with the ligand. We show stable H-bonds and hydrophobic interactions with an occupancy of at least 50%.

The RMSD plot of the protein backbone corresponding to a prototypical MD run, as well as the averaged protein, and the ligand RMSD plots and binding energy timeseries of all five replicas point to a converged dynamics (Figure 5a, Figures S4f–h, and S5f–h). The plot of the total interaction energy for carnosol again displays a fluctuation around a stable average value of about –75 kcal/mol after the initial 5 ns of the MD simulation (Figure 5b).

During the MD simulations, we found that the catechol moieties of carnosol display direct H-bonding interactions with the main chain of Gly60 and the side-chain of Glu37 (Figure 5c). A water-mediated H-bond is also formed between the catechol and Arg68. The ester group of carnosol forms an extensive water-mediated H-bond network with the main chains of Ala11, Cys12, and Pro34, as well as with the side-chains of Lys16, Thr58, and Thr96. Moreover, persistent van der Waals interactions are formed with Val7, Val9, Thr58, Glu37, Gly60, Glu62, Arg68, Tyr71, Met72, and Tyr96. An exhaustive interaction profile averaged across all five replicas for each ligand is presented in Table S6.

The binding free energies calculated using the default LIE parameters (Table 2) show the largest binding affinity for carnosol (ΔG_bind_ = −6.36 kcal/mol), which is in line with the docking results. Carnosic acid does not seem to bind at all, having a positive ΔG_bind_ of 0.16 kcal/mol, whereas rosmanol exhibits a very high relative deviation in the Coulomb contribution, making it hard to quantify its binding potential. 

### 3.5. Factor X Docking

**Figure 6:**
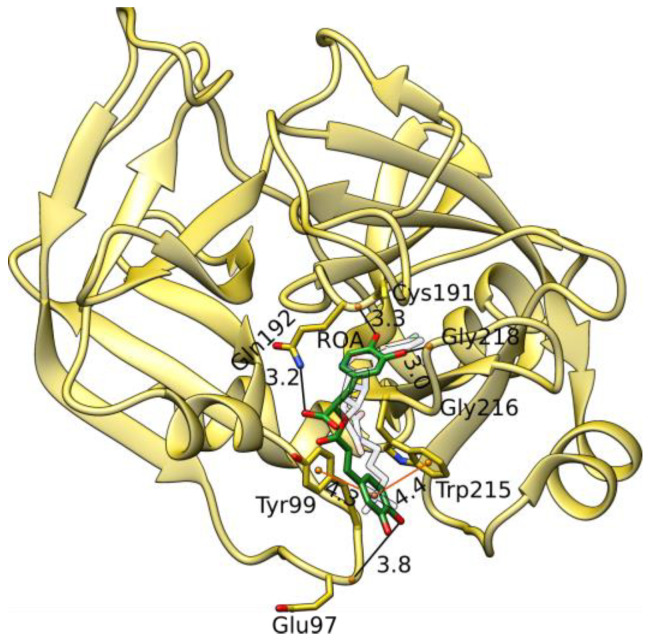
A docked structure of rosmarinic acid (carbon in green sticks) in the factor X binding site (gold cartoons and sticks) (PDB ID 2JKH). H-bonds are shown with black, and π–π interactions are shown with orange lines (distances in Å). Using transparent sticks, we also depict the crystal ligand structure (PDB ID BI7).

Rosmarinic acid docks to the same binding site as the known cationic inhibitors of factor X (for example, ligand PDB ID BI7, protein PDB ID 2JKH) [41] (Figure 6). MD simulations can exhibit a possible dependency of the results on the docking pose used to initiate them [76]. By comparing the docking poses of rosmarinic acid (based on its score, Table S4) with BI7, we find that the third top scoring pose has its center of the caffeic acid catechol ring (Figure 1f) occupying the same location as the cationic center of the crystal ligand BI7 (Figure 6 and Figure S7). This allows for a similar interaction profile in which the catechol ring is embedded between Tyr99 and Trp215 and forms π–π stacking interactions that correspond to the cation–π interactions of the cationic center of BI7. The hydroxyl group of the same catechol ring is concurrently capable of forming a weak H-bond with the backbone of Glu97. Both BI7 and rosmarinic acid also form H-bond interactions with the backbone of Gly216. Rosmarinic acid additionally forms H-bonds with the main chains of Cys191 and Gly218, while the carboxylic acid moiety forms an H-bond with Gln192. Several hydrophobic interactions, especially with Tyr99, Gln192, and Trp215, are also observed.

### 3.6. Factor X MD Simulations and LIE Calculations

**Figure 7:**
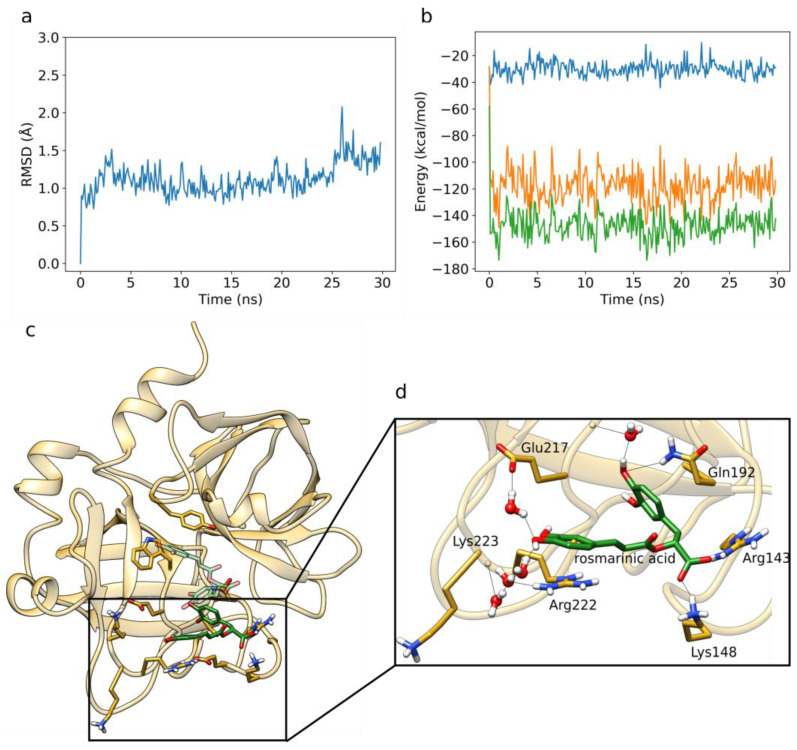
MD simulation of rosmarinic acid bound to factor X. (**a**) RMSD of the protein backbone of the analyzed MD simulation. (**b**) Interaction energy analysis describing the binding between rosmarinic acid and factor X as observed in the MD simulation. (**c**) Rosmarinic acid (carbons depicted in green sticks) occupies a different binding pose compared to the one obtained by molecular docking (carbons shown in transparent green sticks). (**d**) Rosmarinic acid binding within the factor X binding site during the MD simulations. Direct H-bonding interactions are shown with black lines, and the cation–π interaction is shown with an orange line. We display interactions present with an occupancy >50%.

As in the previous simulations, the RMSD plot of the protein backbone corresponding to a prototypical MD run, as well as the averaged protein and ligand RMSD plots and binding energy timeseries of all five replicas, point to a converged dynamics (Figure 7a, Figures S4i, and S5i). The plot of the total interaction energy again displays a fluctuation around a stable average value of about –150 kcal/mol after the initial 5 ns of the MD simulation (Figure 7b).

During the MD simulations, rosmarinic acid samples an alternative pose compared to the one obtained during molecular docking (Figure 7c). This can be explained by the presence of water molecules during the MD simulation, which can strongly affect both the binding pose and the affinity of ligands [77].

Compared to the docked pose, the π–π stacking interactions with Tyr99 and Trp215 are lost and replaced by a direct H-bond with the side-chain of Gln192, as well as by a water-mediated bridge with the backbone of Gly216 (Figure 7d). Direct H-bonds are now formed between the carboxylic acid moiety of rosmarinic acid and the side-chains of Lys148 and Arg143. The catechol ring of the caffeic acid moiety forms a cation–π interaction with Arg222, whereas the catechol hydroxyl groups form water-mediated H-bonding interactions with the backbone of Lys223, as well as the side-chains of Glu217 and Arg222. In addition, van der Waals interactions are formed with Gly218, Lys224, and Glu147. An exhaustive interaction profile averaged across all five is presented in Table S7. We calculated a free energy value of −0.52 kcal/mol for the binding of rosmarinic acid to factor X (Table 3). This suggests that further structural optimization of rosmarinic acid is required for it to be used as a factor X inhibitor; nevertheless, rosmarinic acid may prove to be an interesting starting point for a potential drug design project.

Similar to rosemary diterpenes, rosmarinic acid also undergoes extensive metabolism following oral ingestion. Presumably, it is mostly metabolized by the intestinal microflora [78]. In this process, rosmarinic acid is initially degraded into simpler units, especially caffeic acid, m-coumaric acid, and ferulic acid. Moreover, rosmarinic acid, as well as its listed degraded products, can be sulfated, glucuronidated, and metoxylated in the liver [79,80,81,82,83]. Metabolites identified by the incubation of rosmarinic acid with human liver microsomes in the presence of β-nicotinamide adenine dinucleotide phosphate tetrasodium salt and uridine diphosphate glucuronic acid using glutathione (GSH) as a trapping agent also include glutathione adducts, as well as reduced forms of rosmarinic acid in which one or both catechols are replaced by the quinone rings [84]. Analogous with rosemary diterpenes, due to the large number of possible rosmarinic acid metabolites, and due to the fact that rosmarinic acid has also been detected in its free form following oral administration, we here limited our in silico investigation to rosmarinic acid, but plan to extend it also to the major metabolites in future studies [80,85].

## 4. Conclusions

We present a combined molecular docking and MD study followed by binding free energy calculations to characterize known and potential protein targets of the major rosemary polyphenols. We report that carnosic acid and rosmanol act as viable binders of HIV-1 protease. In addition, carnosol represents a potential binder of the K-RAS oncogene protein. Rosmarinic acid was on the other hand characterized as a weak binder, indicating the need for its further optimization with respect to its applicability as a factor X inhibitor.

We would like to emphasize the importance of considering explicit water molecules in characterizing the binding patterns of ligands. In all the described cases, we observed at least a small change in the interaction patterns during the MD simulations compared to the original docking poses (obtained without water molecules). These changes could be related to the formation of water-mediated H-bonds in the case of the HIV-1 protease and the K-RAS system, where specific polar interactions were replaced by H-bonds bridging one to three water molecules. 

On the other hand, a major change in the binding pose of rosmarinic acid within the factor X binding site was observed, with the new pose stabilized through a water-mediated H-bond network. Due to numerous polar groups present in their structures and due to their tendency for promiscuous binding, such water-mediated hydrogen bonding should indeed represent a general characteristic of polyphenols. Some hints confirming this statement could be obtained by studying polyphenol–protein complexes already deposited in the PDB, such as resveratrol-3-O-glucuronide bound to transthyretin [86], rosmarinic acid bound to myotoxin II [87], or catechol bound to urease [88], all exhibiting water-mediated interactions between the protein and the polyphenolic ligand. Only high-resolution protein structures are useful in this context, as structures solved at a poor resolution tend to lack structural waters and underestimate the internal H-bond networks of the protein [56]. As high-resolution structures of polyphenols bound to proteins are relatively rare in the PDB, MD simulations with explicit water molecules are crucial when studying their interactions, as they facilitate the identification of potential bridging waters that could influence the ligand binding [77].

Last but not least, it is important to emphasize that we limited our study to the free forms of the four investigated rosemary ligands, although such aglycones may not represent the most abundant forms in plasma after oral ingestion due to extensive hepatic and intestinal metabolism of polyphenols in vivo [89,90]. In general, metabolic changes of polyphenols can decrease their bioavailability but, on the other hand, can also give rise to metabolites, which may be more bioactive than the native polyphenols [91]. Such is the case of dihydroresveratrol, formed from resveratrol, which acts as a stronger phytoestrogen than its parent compound [92]. However, we strongly believe that the study of aglycones represents a necessary first step to obtain the basic binding profiles of the investigated ligands, as they represent the central scaffold, from which a drug design campaign could be initiated to optimize the natural compounds. 

Moreover, we compared the high-resolution structure of the aforementioned resveratrol-3-O-glucuronide complex with transthyretin (PDB ID: 5AKS) [86] to the high-resolution transthyretin complex with the free form of resveratrol (PDB ID: 7Q9O) [93], where we can observe an overlapping position of the central aglyconic part of the molecule, while the 3-O-glucuronide forms additional interactions mainly with the surrounding water molecules. Due to the high resolution at which the structures were solved (~1.3 Å), we can also trust with a high confidence the location of the water molecules. We can, therefore, hypothesize that the aglyconic polyphenolic scaffolds bind in a similar manner regardless of their glycosylation. Furthermore, in existing in vivo studies, all rosemary polyphenols are also present in plasma in their free aglyconic form following oral administration [72,73,74,75,80,85]. This fact, in conjunction with the recent surge in the development of alternative pharmaceutical formulations to improve the bioavailability of drugs (e.g., nanoparticles and liposomes), most of which contain the free form of the natural active ingredient [94,95,96,97,98], argues in favor of initially limiting our study to the aglyconic parts of the investigated molecules. However, since we are also fully aware of the potential importance of metabolites present following the oral administration of a plant or a natural product, we plan to extend this study to the corresponding major metabolites in the future.

## Figures and Tables

**Table 1:** The average electrostatic and van der Waals nonbonded contributions of carnosic acid, carnosol, rosmanol, 7O-methylrosmanol, and 7O-ethylrosmanol binding in the complex with HIV-1 protease, along with the corresponding binding free energies calculated using the LIE method.

Ligand/Parameter Set	vdW Contribution (kcal/mol)	Coulomb Contribution (kcal/mol)	ΔG_BIND_ (kcal/mol)
*Carnosic acid*			
α = 0.76, β = 0.24	−7.35 ± 0.63	−1.22 ± 0.17	−8.56 ± 0.76
*Carnosol*			
α = 0.76, β = 0.24	−6.66 ± 1.76	−0.18 ± 1.87	−6.84 ± 0.41
*Rosmanol*			
α = 0.76, β = 0.24	−6.08 ± 1.74	−1.93 ± 1.76	−8.01 ± 1.01
*7O-methylrosmanol*			
α = 0.76, β = 0.24	−4.84 ± 1.56	−2.97 ± 1.95	−7.81 ± 0.75
*7O-ethylrosmanol*			
α = 0.76, β = 0.24	−3.55 ± 0.14	−3.89 ± 0.23	−7.45 ± 0.12

**Table 2:** The average electrostatic and van der Waals nonbonded contributions of carnosic acid, carnosol, and rosmanol to K-RAS binding, along with the binding free energies calculated using the LIE method. The standard LIE α and β parameters obtained from the work of Hansson et al. [48] were applied.

System/Parameter Set	vdW Contribution (kcal/mol)	Coulomb Contribution (kcal/mol)	ΔG_BIND_ (kcal/mol)
*Carnosic acid*			
(a) α = 0.18, β = 0.50	−2.16 ± 0.24	2.32 ± 0.59	0.16 ± 0.41
*Carnosol*			
(a) α = 0.18, β = 0.33	−1.80 ± 0.09	−4.56 ± 0.20	−6.36 ± 0.15
*Rosmanol*			
(a) α = 0.18, β = 0.33	−2.47 ± 0.68	−0.50 ± 3.04	−2.96 ± 2.36

**Table 3:** The average electrostatic and van der Waals nonbonded contributions of rosmarinic acid to factor X binding, along with the binding free energy calculated using the LIE method. The standard LIE α and β parameters obtained from the work of Hansson et al. [48] were applied.

System/Set	VdW Contribution (kcal/mol)	Coulomb Contribution (kcal/mol)	ΔG_BIND_ (kcal/mol)
*Rosmarinic acid*			
(a) α = 0.18, β = 0.50	−2.35 ± 0.22	1.83 ± 0.84	−0.52 ± 0.64

## Data Availability

Data is contained within the article.

## Supplementary Materials

The following supporting information can be downloaded at https://www.mdpi.com/article/10.3390/foods12020408/s1: Table S1. Top three molecular docking scores of carnosic acid, carnosol, and rosmanol with HIV-1 protease; Table S2. The average electrostatic and van der Waals nonbonded contributions of carnosic acid, carnosol, rosmanol, 7O-methylrosmanol, and 7O-ethylrosmanol binding to HIV-1 protease, along with the corresponding binding free energies calculated using the LIE method, with parameters obtained by by Huang et al. (a) and Hansson et al. (b); Table S3. Top three molecular docking scores of carnosic acid, carnosol, and rosmanol with K-RAS; Table S4. Top three molecular docking scores of rosmarinic acid with factor X; Table S5. Interaction frequencies (%) between HIV-1 protease and diterpenic ligands; Table S6. Interaction frequencies (%) between K-RAS and diterpenic ligands; Table S7. Interaction frequencies (%) between factor X and rosmarinic acid; Figure S1. RMSD profiles of ligands in aqueous environment.; Figure S2. Interaction energy profiles of ligands in aqueous environment.; Figure S3. Comparison of binding between the native crystal ligand of HIV-1 protease and carnosic acid; Figure S4. Averaged RMSD profiles across five replicas of the protein and ligand structure; Figure S5. Averaged binding energy profiles across five replicas of ligand to protein binding; Figure S6. Comparison of binding between the native crystal ligand of K-RAS and carnosol; Figure S7. Three best-scoring docking poses of rosmarinic acid.

## Abbreviations

MD	Molecular dynamics
LIE	Linear interaction energy
HIV-1	Human immunodeficiency virus 1
K-RAS	Kirsten-rat sarcoma viral oncogene homolog
RAS/MAPK	Rat sarcoma/mitogen-activated protein kinase
GTP	Guanosine triphosphate
PDB	Protein Data Bank
CHARMM	Chemistry at Harvard Macromolecular Mechanics
CHARMM-GUI	Chemistry at Harvard Macromolecular Mechanics graphical user interface
