# Supplementary material for: Mechanistic Insights of Polyphenolic Compounds from Rosemary Bound to Their Protein Targets Obtained by Molecular Dynamics Simulations and Free-Energy Calculations
Source: Foods. 2023 Jan 14;12(2):408. doi: 10.3390/foods12020408 (PMC9858269; doi:10.3390/foods12020408)
Supplement: Supplementary file 1 [file foods-12-00408-s001.zip › foods-2096275-supplementary.pdf]

Supplementary materials

Table S1. Top three molecular docking scores of carnosic acid, carnosol and rosmanol with HIV-1 protease.

Ligand	Score 1	Score 2	Score 3
Carnosic acid	-21.4106	-21.3113	-21.2960
Carnosol	-22.0796	-21.7720	-21.7070
Rosmanol	-19.0478	-18.6083	-18.5903

Table S2. The average electrostatic and van der Waals nonbonded contributions of carnosic acid, carnosol, rosmanol, 7O-methylrosmanol and 7O-ethylrosmanol binding to HIV-1 protease, along with the corresponding binding free energies calculated using the LIE method, with parameters obtained by by Huang et al. (a) and Hansson et al. (b).

Ligand/ Parameter set	vdW contribution (kcal/mol)	Coulomb contribution (kcal/mol)	ΔG_{BIND} (kcal/mol)
	Carnosic acid		
(a) α = 0.17, β = 0.02	-1.66 ± 0.18	-0.09 ± 0.01	-1.76 ± 0.18
(b) α = 0.18, β = 0.50	-1.76 ± 0.19	-2.34 ± 0.17	-4.10 ± 0.29
	Carnosol		
(a) α = 0.17, β = 0.02	-1.41 ± 0.33	-0.04 ± 0.14	-1.45 ± 0.21
(b) α = 0.18, β = 0.33	-1.50 ± 0.36	-0.66 ± 2.24	-2.16 ± 1.90
	Rosmanol		
(a) α = 0.17, β = 0.02	-1.34 ± 0.40	-0.17 ± 0.15	-1.51 ± 0.28
(b) α = 0.18, β = 0.33	-1.42 ± 0.42	-2.73 ± 2.51	-4.15 ± 2.17
	7O-methylrosmanol		
(a) α = 0.17, β = 0.02	-1.11± 0.40	-0.23 ± 0.15	-1.33 ± 0.26
(b) α = 0.18, β = 0.50	-1.17± 0.42	-3.76 ± 2.47	-4.93 ± 2.07
	7O-ethylrosmanol		
(a) α = 0.17, β = 0.02	-0.80 ± 0.03	-0.30 ± 0.02	-1.10 ± 0.02
(b) α = 0.18, β = 0.50	-0.85 ± 0.03	-5.00 ± 0.29	-5.85 ± 0.26

Table S3. Top three molecular docking scores of carnosic acid, carnosol and rosmanol with K-RAS.

Ligand	Score 1	Score 2	Score 3
Carnosic acid	-20.5045	-19.9349	-19.8753
Carnosol	-23.6509	-23.5006	-22.7920
Rosmanol	-21.5713	-21.5423	-21.4942

Table S4. Top three molecular docking scores of rosmarinic acid with factor X.

Ligand	Score 1	Score 2	Score 3
Rosmarinic acid	-21.4923	-21.123	-20.1228

Table S5. Interaction frequencies (%) between HIV-1 protease and diterpenic ligands. Values larger than 100% are possible because multiple atoms of each aminoacid residue can interact with multiple atoms within the ligand.

Carnosic acid	%	Carnosol	%	Rosmanol	%	7O-methyl rosmanol	%	7O-ethyl-rosmanol	%
ILE47A	1597,1	ILE149B	1265,2	ILE149B	1171,2	ILE149B	1077,7	ILE47A	1575,4
ILE84A	1583,4	ILE47A	1204,2	ILE84A	937,9	ILE47A	1071,7	ILE84A	1434,2
VAL32A	1400,9	ILE84A	1188,5	ILE47A	928,6	ILE50A	1054,1	VAL82A	1362,4
VAL181B	1361,1	VAL181B	936,3	ILE50A	913	ILE84A	1046,6	VAL32A	1130,0
ILE149B	1313,2	VAL32A	923,0	VAL82A	772,8	VAL82A	989,6	ILE54A	1112,0
ARG107B	1194,5	ILE50A	913,1	LEU23A	679,6	PRO81A	792,3	PRO81A	1106,4
ASP29A	1166,4	ILE183B	739,2	VAL32A	647,7	VAL32A	786,1	ILE149B	1049,3
ASP30A	1147,6	PRO180B	729,6	ARG8A	638,2	ILE54A	698,2	THR80A	1010,3
LEU122B	1048,8	ALA28A	687,3	ALA28A	636,8	ALA28A	670,6	ARG8A	867,1
PRO180B	1017,5	ARG107B	666,0	VAL181B	627,7	THR80A	659,4	ALA28A	839,5
ALA28A	985,9	VAL82A	557,9	ILE183B	614,3	ILE183B	577,9	LEU23A	816,2
ILE50A	791,4	LEU122B	549,8	ARG107B	601,2	LEU23A	565,3	ASP25A	782,3
ILE183B	770,6	ASP30A	548,8	ILE146B	545,2	ASP25A	563,6	ILE50A	680,6
LEU76A	715,2	ASP29A	521,7	PRO81A	537,8	GLY48A	529,2	GLY48A	585,4
GLY48A	590,0	GLY48A	513,7	GLY148B	522,7	GLY49A	520,7	ASP30A	494,6
THR31A	515,9	GLY49A	513,6	PRO180B	520,8	ILE153B	493,6	GLY49A	422,8
GLY27A	495,4	PRO81A	428,9	ASP25A	490,7	ARG8A	492,9	GLY148B	350,1
GLY49A	490,2	ILE54A	376,0	ILE153B	477,7	ILE146B	484,6	GLY27A	339,7
ASP25A	354,8	ASP25A	371,7	ALA127B	456,6	GLY148B	471,3	GLY126B	285,1
THR179B	286,3	THR80A	351,4	VAL131B	446,1	VAL131B	464,5	ASP29A	198,3
GLY86A	200,8	GLY148B	330,9	THR179B	445	THR179B	428,6	ALA127B	191,4
VAL56A	135,3	GLY27A	312,3	GLY49A	435,4	VAL181B	414,9	LEU76A	183,0
THR80A	123,8	ILE146B	296,1	GLY126B	402	PRO180B	370,3	GLY147B	166,6
ASP124B	76,5	ILE153B	272,1	GLY48A	396,6	ARG107B	356,3	PRO79A	164,5
ILE54A	64,3	THR179B	271,2	LEU122B	359,4	ASP30A	355,5	THR31A	146,4
GLY150B	64,1	LEU23A	270,0	GLY147B	354,4	ASP124B	354,0	VAL56A	141,7
GLY148B	53,8	LEU76A	249,0	ASP29A	344,9	GLY27A	343,3	ASP124B	122,0
LYS45A	49,2	GLY147B	221,3	THR80A	333,1	GLY147B	303,8	ASP128B	113,9
ILE153B	38,1	ARG8A	206,0	ASP124B	322,9	ALA127B	297,0	ILE146B	97,9
ARG87A	36,5	THR31A	198,6	GLY27A	259,5	LEU122B	274,7	GLY52A	97,7
LEU109B	22,3	VAL131B	182,7	ASP128B	250,9	ASP29A	240,3	GLY51A	87,9
VAL82A	18,6	ASP124B	178,5	ASP30A	212,9	GLY126B	231,4	ILE183B	60,3
PHE53A	16,2	ALA127B	152,1	ILE54A	141,6	PRO178B	149,9	PHE53A	58,6
GLY51A	10,0	GLY126B	116,5	PRO178B	123,1	THR31A	131,8	GLY150B	52,8
ASN182B	9,3	GLY150B	101,6	LEU76A	110,8	LEU76A	126,5	ILE153B	47,4
ILE146B	8,2	PHE53A	98,6	PHE53A	106	GLY51A	101,3	GLY86A	46,1
ILE85A	7,3	GLY86A	84,1	GLY51A	105,2	VAL155B	84,2	VAL181B	44,0
VAL131B	7,2	GLY151B	77,3	VAL155B	71	GLY52A	74,5	PRO180B	43,0
PRO81A	5,8	VAL56A	67,7	GLY150B	60,8	GLY150B	65,3	THR179B	33,8
GLY147B	5,4	PHE152B	62,0	THR31A	50,1	GLY151B	65,2	VAL131B	28,6
MET46A	5,0	ASP128B	52,0	VAL56A	46,6	ASP128B	59,5	GLY78A	25,4
PRO108B	4,9	ARG87A	40,4	LEU109B	46,1	PHE152B	52,5	ARG107B	24,3
GLY52A	4,0	GLY52A	38,6	ARG87A	40,8	PRO79A	51,3	PRO9A	18,8
GLY151B	2,4	ASN182B	36,6	LYS45A	34,5	VAL56A	47,6	ASN83A	17,6
ALA127B	2,1	GLY51A	35,7	MET46A	28,3	GLY86A	40,3	LEU10A	12,8
PRO178B	1,0	LEU109B	34,5	THR125B	25,9	PHE53A	36,4	PHE152B	11,7
GLY126B	0,8	VAL155B	16,7	LEU10A	24,8	GLY177B	35,0	THR125B	10,5
VAL155B	0,4	ASN83A	14,9	ASP129B	23,3	ASN83A	23,5	ARG186B	10,3
PRO79A	0,4	PRO108B	13,5	PRO9A	19,8	ASP129B	18,8	LYS45A	9,4
THR26A	0,2	MET46A	12,6	GLY86A	19,2	LEU175B	16,8	ILE85A	8,2
LEU23A	0,2	PRO178B	12,3	GLY52A	19,1	LEU109B	13,6	GLY151B	8,1
		LYS45A	9,5	GLY177B	18,1	THR125B	13,6	THR26A	5,9
		THR26A	8,4	ARG186B	17,2	PRO9A	10,3	LEU122B	5,3
		LYS154B	8,1	LEU175B	15,8	ARG87A	8,8	ASP129B	3,1
		LEU10A	6,7	PHE152B	15,4	ARG186B	7,0	PRO178B	3,1
		ILE85A	5,6	GLY151B	13	PRO108B	6,7	ILE33A	1,0
		ARG186B	4,6	LEU24A	12,7	LEU10A	6,4	LEU24A	0,8

MET145B	4,0	THR26A	9,4	THR26A	4,9	LYS55A	0,5
LEU175B	3,1	ASN182B	8,3	ASN182B	3,2	VAL155B	0,5
PRO9A	2,4	ASN83A	7,7	ILE85A	3,0	ARG87A	0,3
THR125B	2,1	PRO108B	3,8	LEU24A	2,2	MET46A	0,2
LEU123B	1,7	PRO79A	1,6	GLY78A	2,0	LEU175B	0,2
ASP129B	1,4	ILE85A	1,1	THR130B	1,8	GLY177B	0,2
PRO79A	1,2	ILE132B	0,8	ILE132B	1,6	LYS144B	0,1
GLU133B	0,7	GLY78A	0,6	LEU123B	1,4	ASN182B	0,1
ILE184B	0,5	GLY185B	0,5	MET46A	0,9		
LEU24A	0,3	GLU120B	0,3	LYS45A	0,8		
GLU134B	0,1	THR130B	0,3	LYS154B	0,5		
GLY78A	0,1	ILE33A	0,2	GLY185B	0,3		
ILE132B	0,1	ILE184B	0,1	LYS144B	0,3		
LYS55A	0,1	LEU123B	0,1	ILE33A	0,1		

Table S6. Interaction frequencies (%) between K-RAS and diterpenic ligands. Values larger than 100% are possible because multiple atoms of each aminoacid residue can interact with multiple atoms within the ligand.

**Carnosic acid**	**%**	**Carnosol**	**%**	**Rosmanol**	**%**
ARG68B	1720,2	ARG68B	2342,6	ARG68B	2360,5
TYR96B	1157,4	MET72B	1581,7	TYR96B	1524,5
GLU62B	1090,6	TYR71B	1507,4	MET72B	1447,5
GLN61B	945,9	GLU62B	1399,0	TYR71B	1417,5
THR58B	914,4	THR58B	1284,4	GLU62B	1364,0
CYS12B	803,0	TYR96B	1218,6	THR58B	1359,0
ALA11B	795,7	VAL9B	1183,0	VAL9B	1274,0
ALA59B	684,6	VAL7B	1128,6	CYS12B	969,5
THR35B	663,7	LEU56B	948,2	ALA11B	965,0
GLY60B	654,5	GLU37B	891,4	ALA59B	948,0
PRO34B	619,6	GLY60B	699,6	VAL7B	925,5
LYS16B	594,3	ALA59B	680,0	LYS16B	915,5
GLY10B	512,8	GLN61B	667,7	GLY60B	700,0
VAL9B	480,1	PHE78B	586,9	GLN61B	675,5
HSD95B	474,4	GLN99B	523,4	GLY10B	671,5
GLN99B	430,8	ILE100B	426,5	LEU56B	625,5
MET72B	294,3	GLY10B	393,7	GLN99B	477,5
GLU63B	222,9	GLU63B	383,2	ILE100B	433,5
TYR71B	222,8	ASP69B	378,9	PHE78B	417,0
ASP92B	180,5	VAL8B	216,9	ASP69B	267,5
ARG102B	151,3	ALA11B	197,9	GLU37B	187,0
ASP69B	120,1	MET67B	142,8	VAL8B	185,5
SER65B	108,8	TYR64B	125,0	MET67B	169,5
GLY13B	84,4	LYS16B	115,4	GLU63B	96,5
TYR64B	79,3	CYS12B	113,2	ASP57B	95,0
ILE36B	68,2	HSD95B	110,4	TYR64B	74,5
ILE100B	66,3	ASP57B	82,8	GLY13B	74,0
LYS88B	56,7	GLY75B	47,0	ASP92B	62,0
VAL7B	49,0	ARG73B	38,9	HSD95B	54,0
GLU37B	33,4	GLN70B	20,6	SER65B	30,5
VAL103B	31,5	VAL103B	18,5	ARG102B	26,5
MET67B	23,6	SER65B	15,2	PRO34B	25,5
VAL14B	20,6	ASP92B	7,7	ARG97B	23,5
VAL8B	12,4	ARG102B	3,9	LEU80B	14,0
PHE78B	10,9	THR74B	3,4	VAL103B	10,0
LEU56B	5,1	LYS88B	1,3	GLN70B	10,0
ASP57B	4,5	SER39B	1,2	GLY75B	6,5
GLU98B	3,1	PRO34B	0,9	VAL14B	4,5
ASP33B	2,0	LEU80B	0,3	THR35B	4,0
ALA66B	2,0	THR35B	0,2	LYS88B	3,5
ASN86B	1,5	ASP38B	0,2	ILE93B	2,0
LEU80B	1,3	GLY13B	0,1	ARG73B	1,5

ARG97B	1,0	LYS5B	0,1
SER89B	0,8	LEU6B	0,1
GLN70B	0,8		
ILE93B	0,2		

Table S7. Interaction frequencies between factor X and rosmarinic acid. Values larger than 100% are possible because multiple atoms of each aminoacid residue can interact with multiple atoms within the ligand.

**Rosmarinic acid**	**%**
GLN198A	1498,5
TRP221A	1412,4
GLU223A	1176,4
CYS197A	821,0
CYS226A	792,4
GLU151A	778,8
ARG228A	764,2
LYS152A	751,4
GLY222A	685,3
ARG147A	642,7
GLY224A	641,9
TYR100A	597,4
LYS231A	519,8
SER201A	518,8
ALA196A	468,5
VAL219A	381,0
SER220A	267,2
LYS97A	263,9
HSD57A	258,2
PHE178A	242,5
GLN61A	221,3
GLY233A	217,5
ASP195A	208,3
GLY199A	178,2
PHE41A	150,1
THR99A	141,7
ILE234A	138,3
ASP200A	135,5
CYS42A	107,1
TYR235A	98,4
GLU98A	94,3
TYR60A	80,3
GLN155A	69,7
LYS229A	65,5
ALA227A	49,3
PHE95A	38,1
SER177A	37,4
VAL17A	37,4
TYR232A	32,1
CYS58A	32,1
HSD149A	22,2
GLY202A	11,0
SER176A	9,8
GLY40A	7,4
ALA56A	6,1
ASP103A	5,4
THR96A	4,7
MET184A	3,8
ILE180A	3,7
GLY146A	3,6
VAL142A	2,7
GLY153A	2,0

GLY43A	1,8
ILE179A	1,7
ASP101A	1,0
GLY230A	1,0
ALA55A	0,6
ALA187A	0,5
ALA62A	0,3
GLU194A	0,2
GLU154A	0,2
ASN35A	0,2
ILE16A	0,1

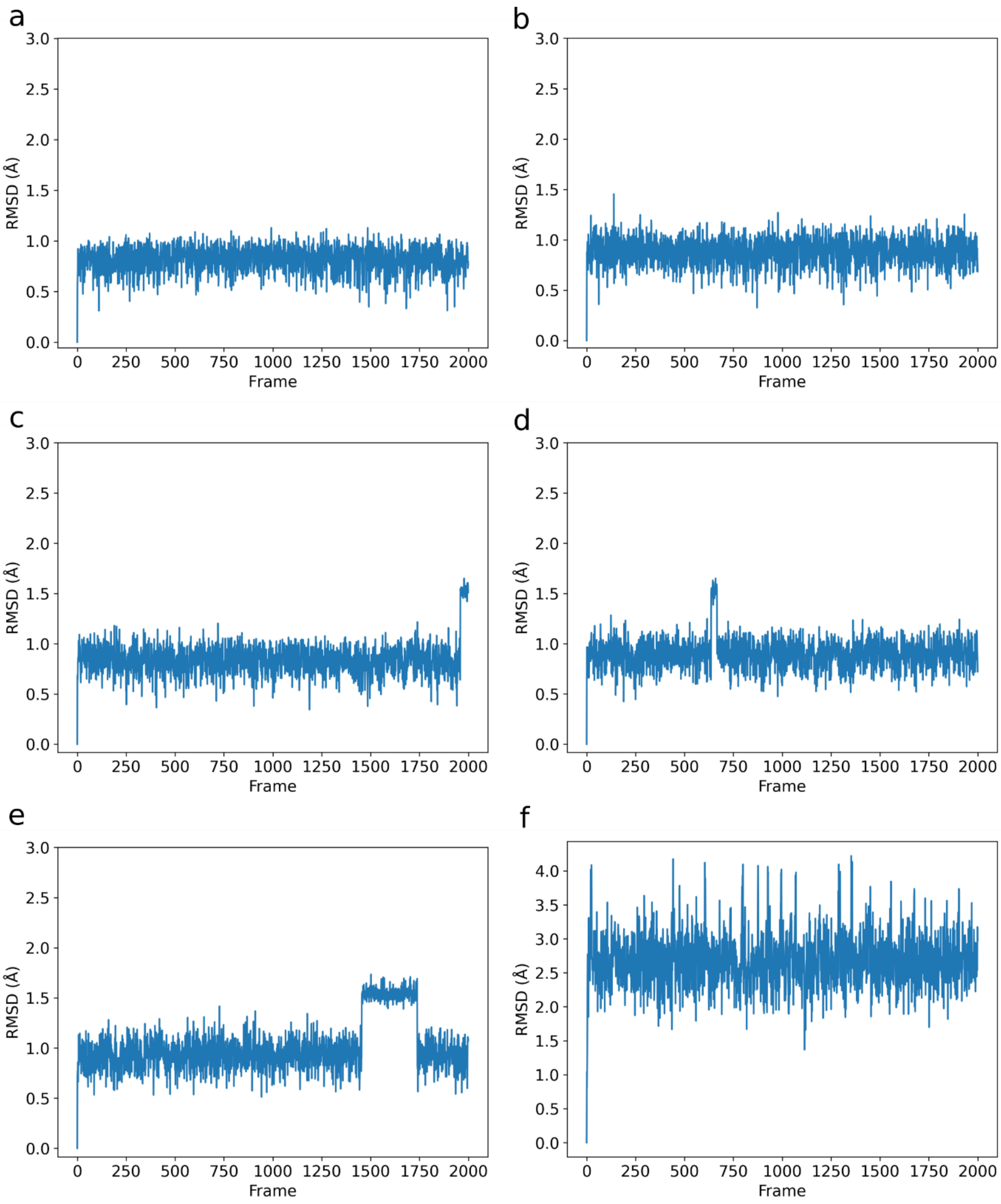

Figure S1. RMSD profiles of ligands in aqueous environment. (a) Carnosic acid, (b) Carnosol, (c) Rosmanol, (d) 7O-methylrosmanol, (e) 7O-ethylrosmanol, (f) Rosmarinic acid.

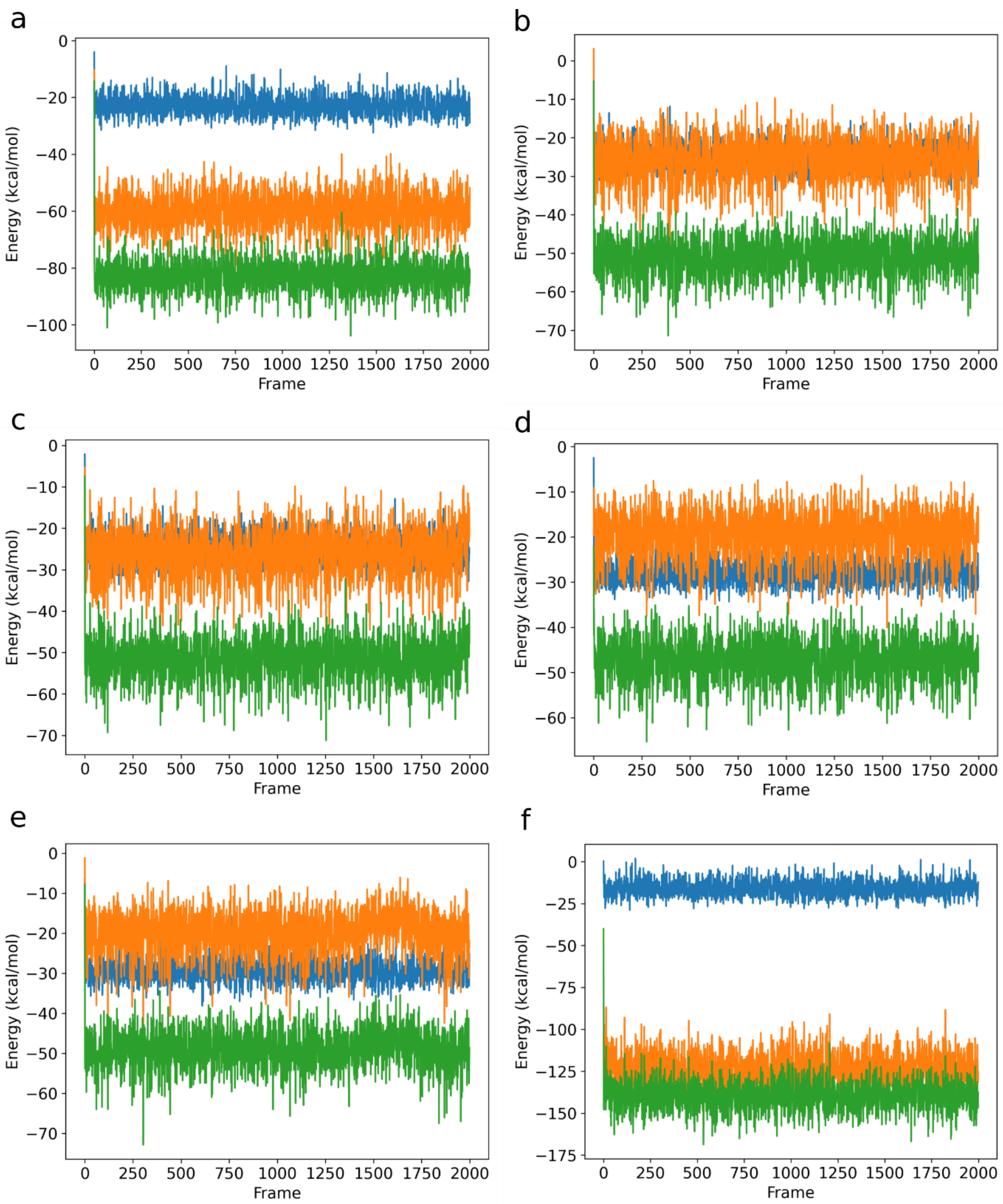

Figure S2. Interaction energy profiles of ligands in aqueous environment. (a) Carnosic acid, (b) Carnosol, (c) Rosmanol, (d) 7O-methylrosmanol, (e) 7O-ethylrosmanol, (f) Rosmarinic acid. The blue line represents the van der Waals contribution, the orange line the electrostatic contribution, and the green line the total interaction energy.

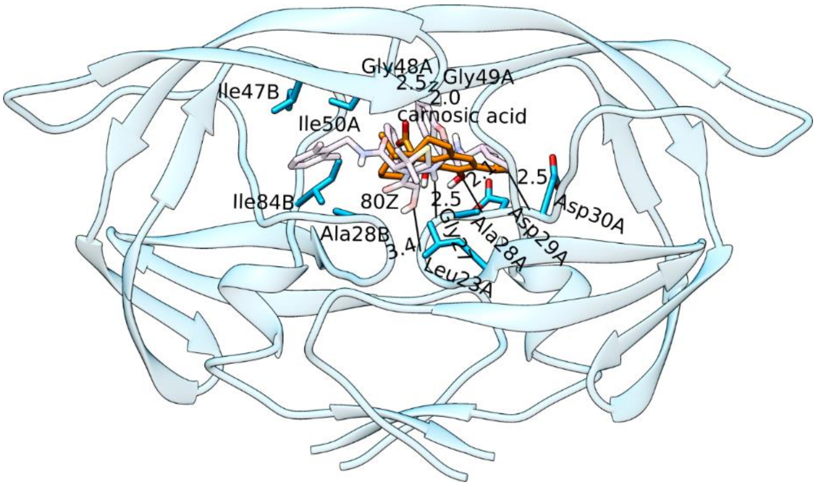

Figure S3. Comparison of binding poses between the native crystal ligand (transparent purple sticks) of HIV-1 protease (PDB ID 5YOK (blue ribbons), PDB Ligand ID 80Z) and carnosic acid (orange sticks). Aminoacid residues forming hydrophobic contacts are shown with blue sticks, whereas H-bond interactions are explicitly shown with black lines. The H-bond distances are measured in Å.

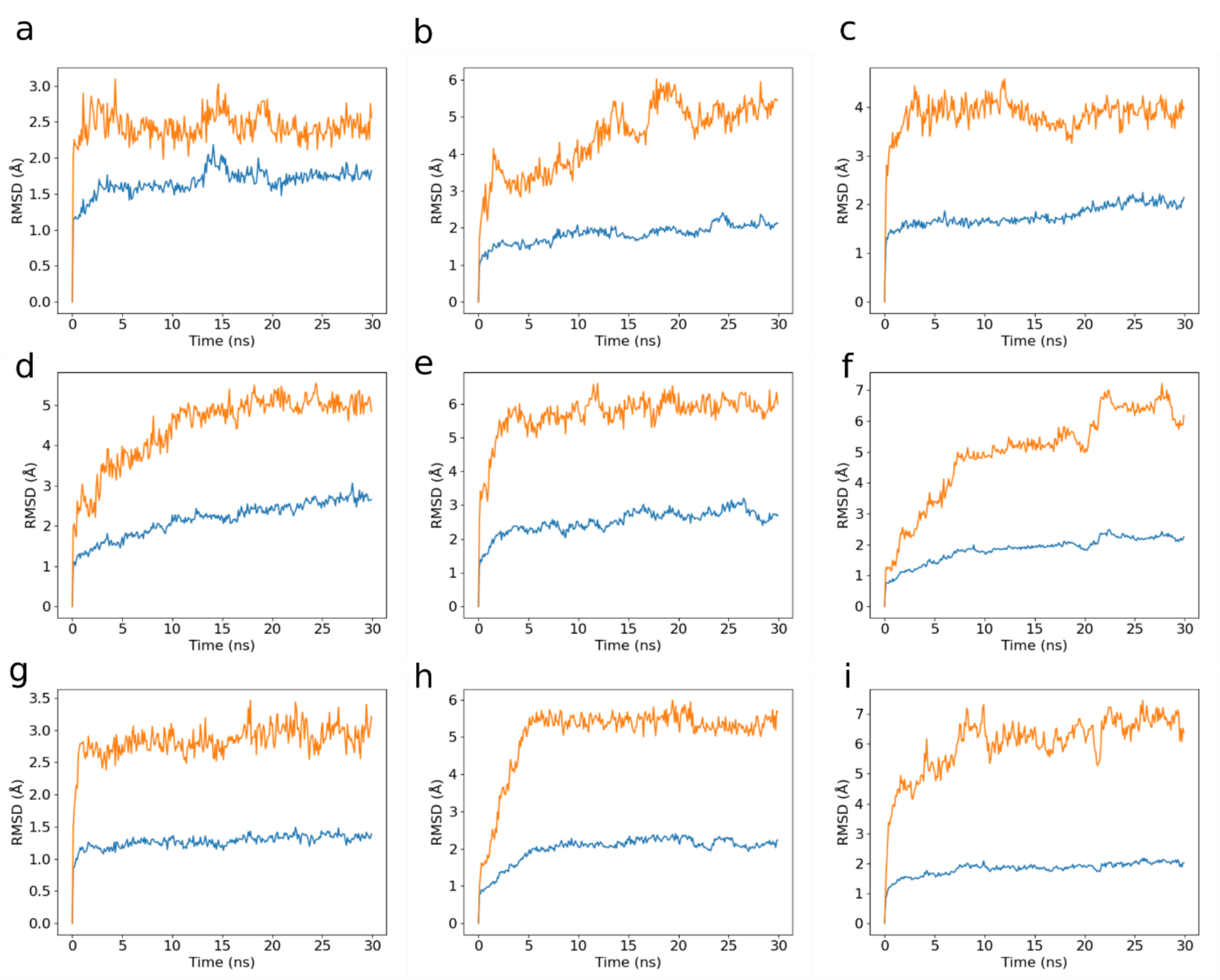

Figure S4. Averaged RMSD profiles across five replicas of the protein and ligand structure. (a) 5YOK-carnosic acid complex, (b) 5YOK-carnosol complex, (c) 5YOK-rosmanol complex, (d) 5YOK-7O-methylrosmanol complex, (e) 5YOK-7O-ethylrosmanol complex, (f) 4LUC-carnosic acid complex, (g) 4LUC-carnosol complex, (h) 4LUC-rosmanol complex, and (i) 2JKH-rosmarinic acid complex. Blue represents RMSD of the protein-ligand structure, whereas the orange for the ligand structure only.

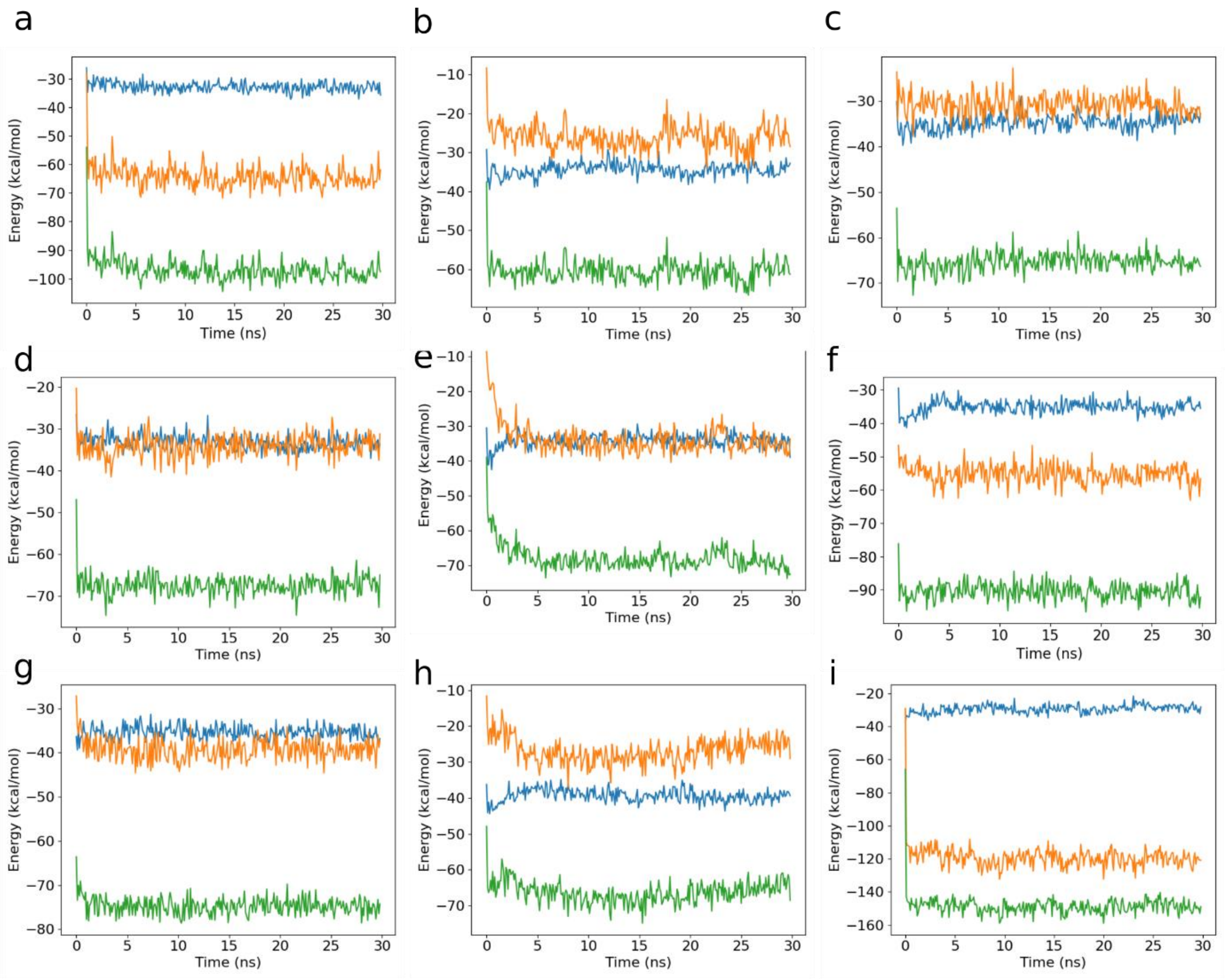

Figure S5. Averaged interaction energy profiles across five replicas of ligand to protein binding. (a) 5YOK-carnosic acid complex, (b) 5YOK-carnosol complex, (c) 5YOK-rosmanol complex, (d) 5YOK-7O-methylrosmanol complex, (e) 5YOK-7O-ethylrosmanol complex, (f) 4LUC-carnosic acid complex, (g) 4LUC-carnosol complex, (h) 4LUC-rosmanol complex, and (i) 2JKH-rosmarinic acid complex. The blue line represents the van der Waals contribution, the orange line the electrostatic contribution, and the green line the total interaction energy.

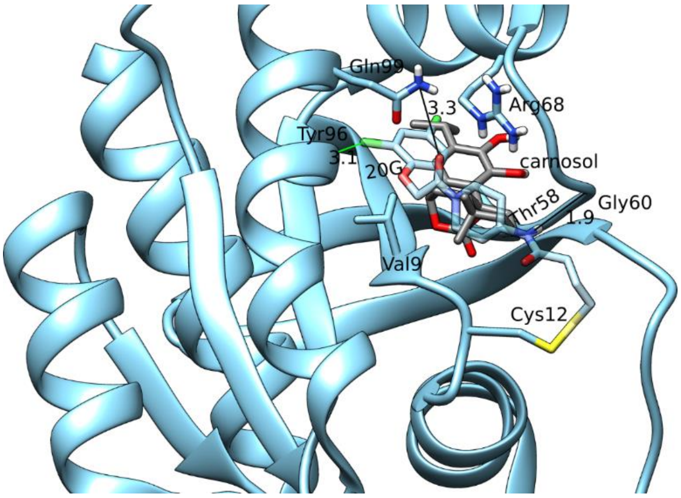

Figure S6. Comparison of binding poses between the native crystal ligand (transparent blue sticks) of K-RAS (PDB ID 4LUC (blue ribbons), PDB Ligand ID 20G) and carnosol (grey sticks). Aminoacid residues forming hydrophobic contacts are shown with blue sticks, whereas H-bond and halogen-bond interactions are explicitly shown with black and green lines, respectively. Their distances are measured in Å.

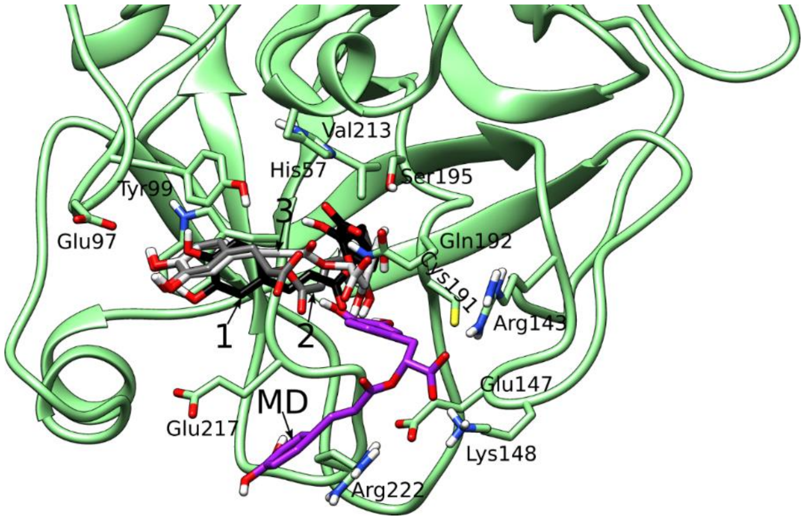

Figure S7. Three best scoring docking poses of rosmarinic acid. Rosmarinic acid with black carbons represents the best scoring pose, with dark grey the second best, and with light grey the third best pose. The latter was selected for subsequent MD simulations. Rosmarinic acid with purple carbons represents a prototypical pose at the end of the MD simulation. Factor X is shown with green cartoons and sticks.
